# Characteristics of GaN-Based Micro-Light-Emitting Diodes for Mbps Medium-Long Distance Underwater Visible Light Communication

**DOI:** 10.3390/nano15171347

**Published:** 2025-09-02

**Authors:** Zhou Wang, Yijing Lin, Yuhang Dai, Jiakui Fan, Weihong Sun, Junyuan Chen, Siqi Yang, Shiting Dou, Haoxiang Zhu, Yan Gu, Jin Wang, Hao Zhang, Qiang Chen, Xiaoyan Liu

**Affiliations:** 1 College of Integrated Circuit Science and Engineering, Nanjing University of Posts and Telecommunications, Nanjing 210023, China; zhouwang@njupt.edu.cn (Z.W.); b22030404@njupt.edu.cn (Y.L.); 2022221004@njupt.edu.cn (Y.D.); b23030720@njupt.edu.cn (J.F.); b23030312@njupt.edu.cn (W.S.); 1023223316@njupt.edu.cn (J.C.); 1224228617@njupt.edu.cn (S.Y.); 1224228412@njupt.edu.cn (S.D.); 1223228218@njupt.edu.cn (H.Z.); yangu@njupt.edu.cn (Y.G.); 2 College of Electronic and Optical Engineering & College of Flexible Electronics (Future Technology), Nanjing University of Posts and Telecommunications, Nanjing 210023, China; jin@njupt.edu.cn; 3 Suzhou Laboratory, Suzhou 215000, China; zhangh@szlab.ac.cn

**Keywords:** gallium nitride, micro-LED, underwater optical wireless communication, long-distance

## Abstract

To promote the development of long-distance high-speed underwater optical wireless communication (UWOC) based on visible light, this study proposes a high-bandwidth UWOC system based on micro-light-emitting-diodes (micro-LEDs) adopting the Non-Return-to-Zero On-Off Keying (NRZ-OOK) modulation. The numerical simulations reveal that optimizing the structural parameters of gallium nitride (GaN)-based micro-LED through dimensional scaling and quantum well layer reduction may significantly enhance optoelectronic performance, including modulation bandwidth and luminous efficiency. Moreover, experimental validation demonstrated maximum real-time data rates of 420 Mbps, 290 Mbps, and 250 Mbps at underwater distances of 2.3 m, 6.9 m, and 11.5 m, respectively. Furthermore, the underwater audio communication was successfully implemented at an 11.5 m UWOC distance at an ultra-low level of incoming optical power (12.5 µW) at the photodetector (PD) site. The channel characterization yielded a micro-LED-specific attenuation coefficient of 0.56 dB/m, while parametric analysis revealed wavelength-dependent degradation patterns, exhibiting positive correlations between both attenuation coefficient and bit error rate (BER) with operational wavelength. This study provides valuable insights for optimizing underwater optical systems to enhance real-time environmental monitoring capabilities and strengthen security protocols for subaquatic military communications in the future.

## 1. Introduction

The escalating demands for marine resource exploitation and advanced oceanographic monitoring have intensified requirements for real-time underwater data transmission, driving substantial research efforts in underwater optical wireless communication (UWOC) paradigms [1,2,3,4,5]. Conventional methodologies employing acoustic and radio-frequency (RF) wave propagation exhibit inherent performance trade-offs [6,7,8]. Acoustic communication suffers from fundamental constraints in low bandwidth and data rates while being capable of kilometer-scale transmission distances, which is due to severe multipath fading and propagation delays [9,10,11,12]. Conversely, RF-based systems demonstrate frequency-dependent attenuation coefficients exceeding 100 dB/m in seawater, with exponential signal degradation at higher frequencies [10,11,12,13], rendering them unsuitable for broadband applications. To overcome the bandwidth limitations of underwater acoustic and RF communication systems and to pursue enhanced data transmission performance, many studies have increasingly focused on other UWOC systems.

Recently, studies have explored laser diode (LD)-based UWOC systems leveraging GHz-level modulation bandwidths, but there still exist several challenges, such as high cost and ocular safety risks [14,15,16,17]. Concurrently, the global transition toward solid-state lighting (SSL) technologies has motivated systematic investigations into light-emitting diode (LED)-based visible light UWOC architectures [18,19,20,21]. These systems offer compelling advantages in cost efficiency, energy conservation, and thermal management, aligning with the operational requirements of sustainable subsea infrastructure [18,19,20,21]. Despite these benefits, conventional broad-area LEDs suffer from intrinsic modulation bandwidth constraints, typically exhibiting −3 dB electrical-to-optical (E-O) bandwidths below 20 MHz due to carrier recombination dynamics and parasitic capacitance effects. To address this limitation, micro-light-emitting-diodes (micro-LEDs) have emerged as disruptive optoelectronic solutions, achieving bandwidth enhancements exceeding two orders of magnitude (>1 GHz) through quantum confinement optimization and reduced RC time constants [22].

To date, the application of micro-LED to the UWOC system was first demonstrated in our previous study in 2017 [23]. It achieved a breakthrough data rate of 800 Mbps across a 0.6 m underwater channel utilizing the Non-Return-to-Zero On-Off Keying (NRZ-OOK) modulation, known as Binary Amplitude-Shift Keying (2ASK) modulation scheme [23,24]. In 2019, subsequent advancements by Arvanitakis et al. proposed a multi-emitter configuration utilizing six series-connected micro-LEDs with wavelength division multiplexing (WDM) technology, attaining an underwater transmission rate of 4.92 Gbps over 1.5 m [25]. However, when the turbidity level increases and the transmission distance extends, the communication performance of micro-LEDs-based UWOC will decline significantly. More recently, Lin et al. developed a bidirectional UWOC system at a depth of 2.3 m employing indium gallium nitride (InGaN) micro-LED arrays in 2021, achieving system-level integration of high-speed communication with energy harvesting functionality. Despite these innovations, critical challenges persist in circuit impedance matching and adaptive equalization for mitigating inter-symbol interference in turbid channels. The comparative analyses of reported LED-based UWOC systems are summarized in Table 1.

In this study, we proposed an effective UWOC system based on GaN micro-LED, achieving long-distance communication at a high data rate utilizing the NRZ-OOK modulation. To obtain optimal communication performance, the characteristics of the GaN-based micro-LEDs with various sizes and different quantum well cycles have been studied and compared through numerical simulations in SILVACO 2018 software. Furthermore, the UWOC performance of the system under different transmission distances was systematically studied. The proposed long-distance UWOC system successfully achieved underwater audio communication at an 11.5 m underwater communication distance with a low light output power of 12.5 μW and a low bit error rate (BER) of 2.7 × 10^−3^. The study shows that the UWOC system based on micro-LEDs has great potential in long-distance wireless optical communication.

## 2. Simulations and Experiments

### 2.1. Model of Micro-LED Structure

**Figure 1:**
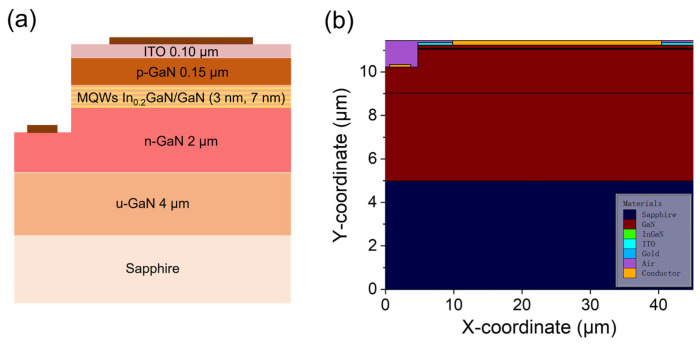
Structure of GaN-based micro-LED: (**a**) schematic diagram (not to scale); (**b**) simulation structure diagram.

Figure 1 illustrates one kind of the simulated structures of the GaN-based blue micro-LED designed for systematic optoelectronic performance evaluation as an example. The device features a 40 µm × 40 µm active region with five-periods InGaN/GaN multiple quantum wells (MQWs) structure. The sample consists of a 4 μm thick undoped GaN layer, a 2 μm thick n-type GaN layer (Si-doping = 5 × 10^18^ cm^−3^), an active region that contains five pairs of a 3 nm thick In_0.1_GaN well layer and a 7 nm thick GaN barrier layer, and a 0.15 μm thick p-type GaN layer (Mg-doping = 10^20^ cm^−3^). The epitaxial layers are on a c-plane sapphire substrate. To investigate dimensional and quantum confinement effects, we perform numerical simulations using Silvaco TCAD software. Four device sizes (10–40 μm edge length, the step is 10 μm) and five MQWs configurations (1–5 cycles, the step is 1 cycle) were compared, which are major factors of the optoelectronic device. The simulation employed calibrated physical models, including Fermi-Dirac carrier statistics, Shockley–Read–Hall recombination, and photon density-dependent radiative recombination mechanisms.

### 2.2. Model of UWOC System Channel

**Figure 2:**
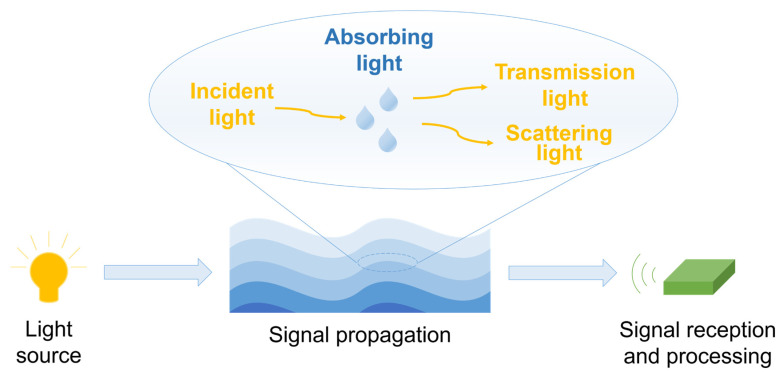
Principles of UWOC systems and optical properties of water.

The UWOC system can be divided into four parts, which are light source, signal propagation, signal reception, and signal processing, as shown in Figure 2. The optical signal will be captured by the signal receiving end, such as PIN photodetector (PIN) or avalanche photodetector (APD). Additionally, the optical signal will be converted into an electrical signal. Then, the electrical signal is filtered and amplified, and finally the original information of the communication is restored.

The most common light sources of UWOC are LEDs and LDs. The light sources emit light signals underwater, and the propagation of light signals is related to the optical properties of water. When light propagates through water, some of its energy is absorbed and scattered by water itself and particles in it [2].

Specifically, the absorption effect of the underwater environment reduces the propagation distance of light, while the scattering effect affects the propagation direction of light, which increases the BER of the signal [28] and affects the communication performance of UWOC. Due to different underwater environments, water turbidity, salinity, biodiversity, and other related factors have different effects on the attenuation of light. To further verify the power attenuation characteristics of visible light signals in UWOC, we use MATLAB R2020a to construct the UWOC channel model under pure seawater. The simulation system can be divided into three modules including emitter, channel, and receiver. The emitter adopts NRZ-OOK modulation to generate the signal source and encode the information. The optical wave power attenuation model in pure seawater is established, considering factors such as salinity and wavelength of incident light. The receiver demodulates the signal and calculates the BER.

According to the absorbance-defining equation, one can list the expression for the absorption coefficient *α*(*λ*), where *λ* is the wavelength of the incident light, ∆*D* is the length of the water tank, *P_I_*(*λ*) is the power of incident light, *P_A_*(*λ*) is the absorbed light power of the water [28].

(1) $$\alpha (\lambda )=\underset{\Delta D\to 0}{\lim}\frac{P_{A}(\lambda )}{P_{I}(\lambda )\cdot \Delta D}$$

From the definition of scattering degree, the expression of the scattering coefficient *β*(*λ*) can be listed as follows, where *P_S_* is the scattered optical power [28].

(2) $$\beta (\lambda )=\underset{\Delta D\to 0}{\lim}\frac{P_{S}(\lambda )}{P_{I}(\lambda )\cdot \Delta D}$$

In addition, the geometric attenuation of the light source is also an important influencing factor. The emission aperture of micro-LED is very small relative to the transmission distance. Therefore, it can be approximately regarded as an ideal point light source, and the geometric attenuation factor *σ* can be expressed as $\sigma ={\left(\frac{D}{2L\times \tan\theta }\right)}^{2}$. Here, *D* represents the diameter of the aperture of detector, *L* represents the transmission distance, *θ* represents the divergence angle of the light beam. Based on the absorption and scattering coefficients, it is possible to define the total attenuation coefficient *η*(*λ*), which means the ratio of the lost optical power to the incident optical power for water depth *H* (*H* tends to 0). The expression for the total attenuation coefficient is as follows, in units of m^−1^ [28].

(3) η(λ)=σ(α(λ)+β(λ))

The simulation results of the total attenuation coefficient can be obtained by associating functions (1), (2), and (3) and substituting the simulation parameters. The specific data are stored in Supporting Information, Table S1. In summary, these coefficient functions are counted for evaluating the total attenuation coefficient, providing a reference index for designing a rational UWOC system.

### 2.3. Practical Experiments of the UWOC System

**Figure 3:**
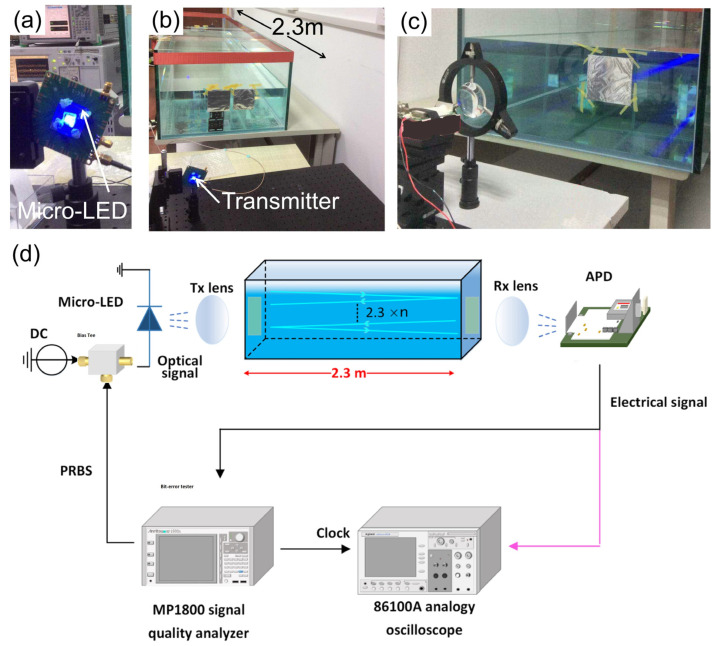
Pictures of the experimental setup and schematic diagram: (**a**) the packaged micro-LED; (**b**) the tank to simulate underwater channels; (**c**) the avalanche photodiode, the lens, and reflector on tank side wall; (**d**) schematic diagram of the proposed micro-LED-based real-time UWOC system and underwater audio communication. *n* stands for the reflection times of the light beam.

The UWOC system employed a single micro-LED from the monolithic 4 × 4 GaN-based blue micro-LED array, with the wafer epitaxial structure grown on the c-plane sapphire substrate. The manufacture process of this specific device can be found in Supporting Information, Figure S1. The schematic diagram of micro-LED-based real-time underwater audio communication using NRZ-OOK modulation is illustrated in Figure 3.

Figure 3a displays a packaged GaN based blue micro-LED. Figure 3b exhibits the transmitter and a light propagation channel for UWOC with water in the tank. And the communication link consists of an APD receiver with a focus lens, as shown in Figure 3c. Figure 3d illustrates the architecture of a real-time transmission system employing a visible-light-communication (VLC) line-of-sight (LOS) link. The system integrates a GaN-based micro-LED array as the optical transmitter modulated with pseudorandom binary sequences (PRBS) signal from a MP1800 signal quality analyzer, coupled with an aspheric collimating lens (Tx lens) to minimize beam divergence. The collimated transmitted signal propagates through a water tank engineered with high-reflectivity sidewall mirrors, effectively extending the underwater transmission length to 11.5 m through reflections. The transmitted signal was focused on an APD using a receiver lens (Rx lens), and the optical signal was converted into electrical signal for communication performance measurement. The data transmission rate and BER were monitored with an 86100A analogy oscilloscope from Agilent Technologies, Santa Rosa, CA, USA and MP1800 signal quality analyzer from Anritsu Corporation, Atsugi, Kanagawa Prefecture, Japan.

## 3. Results and Discussions

### 3.1. Results of Micro-LED Simulation

**Figure 4:**
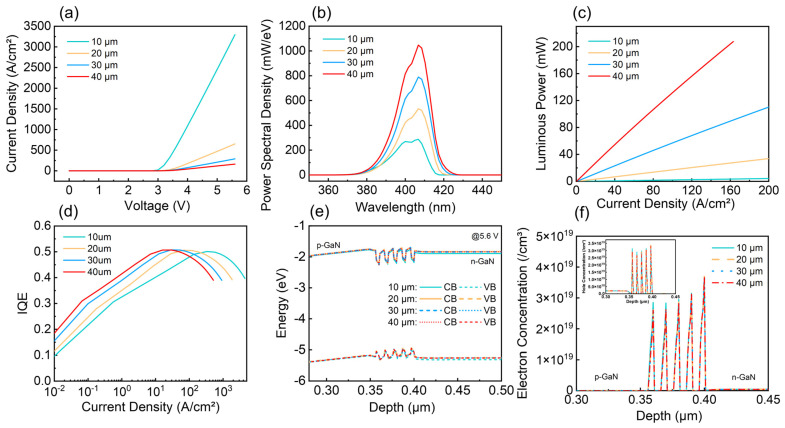
Simulated optoelectronic characteristics of micro-LEDs with different sizes: (**a**) The curves of current density varying with voltage (J-V); (**b**) EL spectra at a voltage of 5.6 V; (**c**) The curves of luminous power varying with current density (L-J); (**d**) IQE; (**e**) energy band gap diagram at the quantum wells (5.6 V bias); (**f**) carrier concentration distribution (5.6 V bias).

There have been numerous studies focusing on the size-dependent effects of GaN/InGaN micro-LEDs. As the device size of micro-LEDs becomes smaller, many of the optoelectronic characteristics will change. These changes can be observed in current-versus-voltage characteristics, light output power, electroluminescence (EL) spectra, quantum efficiency, and so forth. Here, we simulated the optoelectronic characteristics of micro-LEDs with sizes from 10 µm to 40 µm, as shown in Figure 4. The current density of micro-LED decreases as the device size increases under the same forward bias in Figure 4a. This is mainly because the smaller devices exhibit shorter lateral current paths, leading to more uniform current distribution. As a result, for the same applied voltage, a higher current flows through the smaller-area device, resulting in a larger calculated current density [29]. Figure 4b,c illustrate the power spectral density at a voltage of 5.6 V and the luminous power as a function of current density for micro-LEDs of varying sizes. As the device size decreases, the surface-to-volume ratio increases, making the influence of surface-related defects in the active region more significant. These defects introduce surface states that act as non-radiative recombination centers, capturing electrons and holes. Consequently, the proportion of non-radiative recombination increases, reducing EL intensity and luminous power. Figure 4d shows the curve of internal quantum efficiency (IQE) of micro-LED versus the current density. The lower peak IQE for micro-LED with smaller device size results from more non-radiative recombination caused by sidewall defects under lower current density. And the peak IQE occur at a higher current density, which is mainly due to the better current spreading capabilities [30]. In addition, Figure 4e shows the conduction band (CB) and the valence band (VB) in the active region for micro-LED with different sizes under the 5.6 V bias condition. With the increase of size, the band gap changes slightly (40-μm size: 2.700 eV; 30-μm size: 2.700 eV; 20-μm size: 2.701 eV; 10-μm size: 2.753 eV). The corresponding carrier concentration distributions are depicted in Figure 4f, indicating that electrons and holes are relatively uniformly distributed in micro-LED.

**Figure 5:**
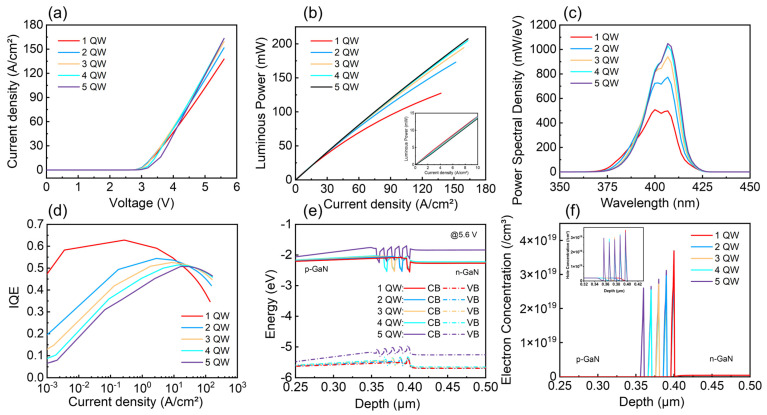
Simulated optoelectronic properties of micro-LEDs with different quantum well cycles: (**a**) J-V curves; (**b**) L-J curves; inset is a magnified view under low current densities from 0–10 A/cm^2^. (**c**) EL spectra at a voltage of 5.6 V; (**d**) IQE curves; (**e**) energy band gap diagram at the quantum wells (5.6 V bias); (**f**) carrier concentration distribution (5.6 V bias).

Figure 5 shows the results of simulating optoelectronic characteristics of micro-LED with different numbers of quantum wells. As shown in Figure 5a, it can be seen that the current density of micro-LEDs with fewer quantum wells is relatively higher under low bias, mainly due to their higher average carrier concentration. Conversely, micro-LEDs with more quantum wells have a higher current density at high voltage bias, which can be attributed to the higher probability of radiative recombination with multiple quantum wells. These trends are consistent with the luminous power curve demonstrated in Figure 5b, and the inset in Figure 5b shows a magnified view of low current densities ranging from 0 to 10 A/cm^2^. Figure 5c shows the EL spectra simulation curve of the micro-LED under an applied voltage of 5.6 V. When the quantum well number increases, the power spectral density of the device increases, owing principally to the enhanced radiation recombination and reduced Auger recombination. Figure 5d displays the IQE versus current density. It can be seen that under low current density injection, the peak IQE increases as the quantum well number decrease. This is because micro-LEDs with more quantum wells suffer from insufficient carrier injection, resulting in lower radiative recombination, whereas fewer quantum well devices exhibit effective carrier injection, enhancing radiative recombination [31]. Under high current density injection, the efficiency of micro-LEDs with fewer quantum wells declines more rapidly, primarily resulting from significant carrier overflow and high Auger recombination. The efficiency droop can be explained by the ABC model, wherein the Auger recombination mechanism becomes more dominant under high current density, generating significant lattice heat and consequently reducing the recombination efficiency of micro-LEDs. Figure 5e presents the energy band diagram of the micro-LED with various pairs of quantum wells under an applied voltage of 5.6 V, while the associated carrier concentration distributions are illustrated in Figure 5f. At high current density, as the quantum well number increases, electrons and holes have a higher chance of being captured by one of the multiple quantum wells. This distribution characteristic enhances the radiation recombination in each quantum well to tend towards equilibrium, thereby making radiative recombination more concentrated and enhancing the radiative luminescence intensity at high current density injection. Therefore, the micro-LED with the size of 40 μm × 40 μm featured 5 periods of MQWs has the best luminous performance for applications in the UWOC system and can be selected as the emitter for the experiment.

### 3.2. Results of UWOC System Channel Simulation

Since the current operating wavelength of UWOC generally ranges from 400 nm to 580 nm, we select five incident light wavelengths of 440 nm, 475 nm, 490 nm, 505 nm, 515 nm, and 525 nm and carry out the simulation experiments at a communication distance of 100 m. The experimental results are shown in Supporting Information, Table S1. The signal-to-noise ratio (SNR) is lower, whereas the BER is higher for micro-LED with longer emission wavelengths. This phenomenon indicates the reduction of communication quality with a greater wavelength of incident light. Although the BER increases constantly with greater wavelength, it is still less than the threshold 3.8 × 10^−3^ shown in Supporting Information, Figure S2, which is a good sign. Eventually, the micro-LED with a 440 nm wavelength was selected as the emitter for the subsequent experiment due to its attenuation coefficient and BER being the lowest, which demonstrates a great potential in the UWOC system.

### 3.3. Results of Practical Experiments of UWOC System

**Figure 6:**
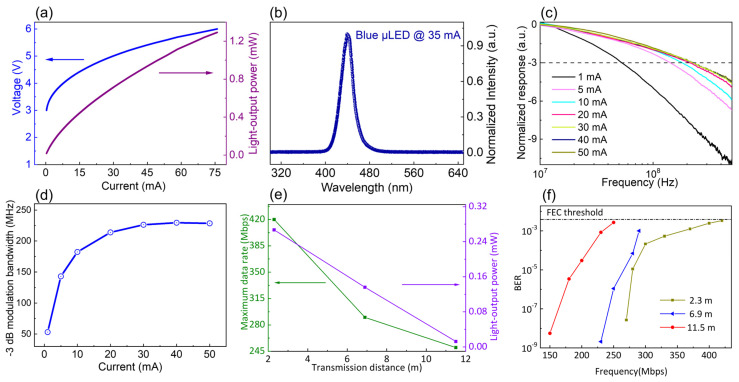
(**a**) I-V and L-I characteristics of the 40 μm × 40 μm blue micro-LED; (**b**) EL spectra at 35 mA injection current for the micro-LED; (**c**) normalized frequency response and (**d**) extracted −3 dB modulation bandwidth of the 40 μm × 40 μm micro-LED at different injection currents from 1 mA to 50 mA. The −3 dB bandwidth label is marked in the dashed line; (**e**) maximum achievable data rate and received light-output power as a function of transmission distance; (**f**) BER versus data rate at different underwater transmission distances. The dash line is the FEC threshold limit labeled.

The current-versus-voltage (I-V) and light-output power (L-I) characteristics of the micro-LEDs are shown in Figure 6a. Figure 6b shows the EL spectra of the micro-LED at a 35 mA injection current, which is measured with an Ocean Optics USB4000 Spectrometer. It can be seen that the peak emission wavelength is around 440 nm with full-width at the half-maximum (FWHM) of ~29 nm. Notably, the 440 nm blue micro-LED is more suitable for low-loss underwater transmission (see Figure S2) [23,25,32]. Figure 6c illustrates the normalized frequency response of micro-LED at different injection currents. The extracted −3 dB modulation bandwidth is shown in Figure 6d. As the injection current increases, the −3 dB modulation bandwidth of the micro-LED increases, apparently saturating at around 40 mA of injection current. This is because the increase of injection current improves the carrier concentration and the electron-hole recombination rate, resulting in improving modulation bandwidth performance of the micro-LED.

As is shown in Figure 6e, the UWOC system can achieve the maximum data rate of 420 Mbps under short communication distances of 2.3 m. Also, it can be found that when the communication distance increased up to 6.9 m, the maximum achievable data rate of the UWOC system is 290 Mbps. However, when the communication distance rises to 11.5 m, the UWOC can achieve the maximum data rate of 250 Mbps. The main reason for the reduction of maximum data rate with the increase of communication distance is the decrease of incoming optical power (IOP) received by the photodetector under longer communication distance, as seen in Figure 6e. The IOPs received by the photodetector under communication distances of 2.3 m, 6.9 m and 11.5 m are 0.267 mW, 0.136 mW and 0.0125 mW, respectively. This is of great significance for realizing long-distance transmission of micro-LED-based underwater communication systems.

The measured BERs as a function of data rate at various underwater communication distances are shown in Figure 6f. The dashed line represents the FEC threshold of 3.8 × 10^−3^. To extend the light propagation distance, high-reflectivity reflectors are employed to reflect the light beam within the 2.3 m water tank. The high-reflectivity reflectors can be seen in Figure 3b,c, which are the reflector mirrors attached to tank side-walls with aluminium foil. It can be seen that the BER raised when we increased the transmission data rate. The maximum data rate declined when the underwater transmission distance was extended. Under the FEC threshold, the maximum achievable data rate of up to 420 Mbps, 290 Mbps, and 250 Mbps were obtained at the distances of 2.3 m, 6.9 m, and 11.5 m. The low data rate at a communication distance of 11.5 m is attributed to the reduction in incoming optical power, resulting from the expansion of the light beam spot size during propagation and the increased light attenuation over longer distances underwater [33].

Table 2 summarizes the maximal achievable data rates and the corresponding BERs at different UWOC distances. To assess the applicability of the 440 nm blue micro-LED-based UWOC system, the underwater communication distances were increased from 2.3 m, 6.9 m, and 11.5 m. A data rate of up to 420 Mbps with the BER of 3.4 × 10^−3^ was obtained within a 2.3 m UWOC link. When the underwater distance was extended to 11.5 m, a 250 Mbps data rate was achieved with the lowest received optical power. Compared to the previous reported UWOC system [23,25,26,27], a record micro-LED-based UWOC distance over 11.5 m with a real-time data rate of 250 Mbps has been experimentally demonstrated. Four reflections of the light beam were realised across the 0.6 m width of the water tank to achieve an 11.5 m communication distance.

To investigate the power attenuation of the UWOC system, we collimated the light beam underwater and measured the incoming optical power in air and under water at a middle distance of 6.9 m. The IOP in air was 0.334 mW. The light-output power under water was 0.136 mW. The optical attenuation coefficient of the micro-LED was ~0.56 dB/m, indicating that the light was indeed absorbed and scattered by water molecules and the water tank. The relatively lower attenuation provides reference for tuning optical antennas applied as receiver in UWOC system.

**Figure 7:**
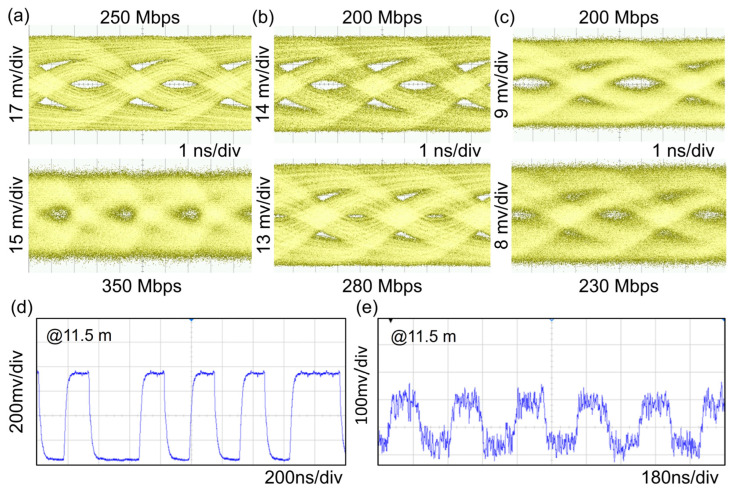
Eye diagrams versus data rate at different underwater distances of (**a**) 2.3 m at data rates of 250 Mbps and 350 Mbps, (**b**) 6.9 m at data rates of 200 Mbps and 280 Mbps, and (**c**) 11.5 m at data rates of 200 Mbps and 230 Mbps. Experimental testing of the audio transmission based on a micro-LED UWOC system received signal for the optical wireless link at an underwater distance of 11.5 m, (**d**) emitted, and (**e**) received signal.

Figure 7a–c compare the eye diagrams of the micro-LED-based UWOC at various transmission distances of 2.3 m with data rates of 250 Mbps and 350 Mbps, 6.9 m with data rates of 200 Mbps and 280 Mbps, and 11.5 m with data rates of 200 Mbps and 230 Mbps. From the horizontal comparison, the eye diagram is gradually blurred as the communication distance increases. The result indicates that extending the communication distance degraded signal quality and potentially increased signal interference. A vertical analysis revealed that raising the data transmission rate also caused the eye diagram to close. This is likely due to the data transmission rate exceeding the current capacity of the UWOC system. Subsequent experiments are planned to enhance signal transmission quality by modulating the data rate to adjust the transmission capacity of the UWOC system or by replacing the receiver to improve the accuracy of data reception.

To verify the performance of the UWOC system, audio signals were transmitted using a mobile phone. The transmitted audio signal waveforms were captured with a high-speed oscilloscope. The received modulated audio signal was then demodulated and converted back into an audio signal using an analog-to-digital converter module with a USB 2.0 interface. Subsequently, the audio signal was played in real-time through a Bluetooth speaker. Figure 7d,e illustrate the waveforms of audio signals before and after transmission through the optical wireless channel at an underwater distance of 11.5 m. It is evident that real-time underwater audio signal communication was successfully accomplished at a distance of 11.5 m with a minimum optical input power of 12.5 μW (Figure 6e). The transmitted signal is distorted more severely compared to the pre-transmission signal. To reduce signal distortion, we are ready to explore new modulation techniques, such as Orthogonal Frequency-Division Multiplexing (OFDM) and Pulse Position Modulation (PPM). Furthermore, we could improve the luminous efficacy and bandwidth performance of micro-LEDs. In addition, employing a specialized optical lens to decrease the divergence angle of the incident light is another method under consideration to reduce light loss.

## 4. Conclusions

In summary, this study demonstrates a UWOC system based on high-bandwidth micro-LEDs, which adopts the NRZ-OOK modulation scheme and realizes long-distance communication of more than 10 m with high speed. Through systematic Silvaco TCAD simulations and experimental validation, we identified an optimized micro-LED architecture featuring a 40 × 40 μm^2^ active region with five pairs of InGaN/GaN QWs. The experimental characterization indicated that real-time data rates of 420 Mbps, 290 Mbps, and 250 Mbps were attained at underwater communication distances of 2.3 m, 6.9 m, and 11.5 m, respectively. Additionally, underwater real-time audio signal communication was successfully achieved at a distance of 11.5 m with a minimum optical input power of 12.5 μW. Moreover, the optical attenuation coefficient of the micro-LED was ~0.56 dB/m, which was small enough to realize a long-distance high-speed UWOC system. This study also examined how the attenuation coefficient varies with changes in the wavelength of incident light in pure seawater through channel simulations, providing precise parameters, i.e., SNR and BER, showing enhancement in communication quality as the wavelength of incident light decreases. Overall, this study provides reference for the attenuation of light with the extension of transmission distance in long-distance UWOC system. The advancement is conducive to promoting development across multiple fields such as marine science and resource exploitation, providing crucial support for related research and industrial activities.

## Figures and Tables

**Table 1:** The communication performance summaries of LED-based UWOC system.

Year	Type of LED	Size	Modulation Scheme	Channel Length	Data Rate	BER	Ref.
2017	Blue GaN ^a^ micro-LED	80 µm	NRZ-OOK ^b^	0.6 m	800 Mbps	1.3 × 10^−3^	[23]
2019	Array of six micro-LED pixels	80 μm	OFDM ^c^	4.5 m	3.4 Gpbs	3.1 × 10^−3^	[25]
2021	Green InGaN micro-LED	80 µm	NRZ-OOK	2.3 m	660 Mbps	3.3 × 10^−3^	[26]
2022	Blue mini-LED	175 µm	PAM-4 ^d^	2 m	4.4 Gbps	2.8 × 10^−3^	[27]
2025	Blue GaN micro-LED	40 µm	NRZ-OOK	11.5 m	250 Mbps	2.7 × 10^−3^	This study

^a^ GaN represents Gallium Nitride. ^b^ NRZ-OOK represents Non-Return-To-Zero On-Off Keying. ^c^ OFDM represents Orthogonal Frequency-Division Multiplexing. ^d^ PAM-4 represents 4-ary Pulse Amplitude Modulation.

**Table 2:** Performance of UWOC based on a 440 nm blue micro-LED using NRZ-OOK modulation.

Distance (m)	Incoming Optical Power (mW)	Maximum Data Rate (Mbps)	BER
2.3	0.267	420	3.4 × 10^−3^
6.9	0.136	290	1.0 × 10^−3^
11.5	0.0125	250	2.7 × 10^−3^

## Data Availability

The original contributions presented in this study are included in the article. Further inquiries can be directed to the corresponding authors.

## Supplementary Materials

The following supporting information can be downloaded at https://www.mdpi.com/article/10.3390/nano15171347/s1: Table S1: Results of total attenuation coefficient versus wavelength for pure seawater at 100 m communication distance; Figure S1: The typical manufacturing process of GaN-based micro-LED; Figure S2: Variation of total attenuation coefficient with wavelength for pure seawater at 100 m communication distance.
