# Supplementary material for: Characteristics of GaN-Based Micro-Light-Emitting Diodes for Mbps Medium-Long Distance Underwater Visible Light Communication
Source: Nanomaterials (Basel). 2025 Sep 2;15(17):1347. doi: 10.3390/nano15171347 (PMC12430584; doi:10.3390/nano15171347)
Supplement: Supplementary file 1 [file nanomaterials-15-01347-s001.zip › nanomaterials-3704749-supplementary.pdf]

nanomaterials

Supplementary Material

Characteristics of GaN-Based Micro-Light-Emitting Diodes for Mbps Medium-Long Distance Underwater Visible Light Communication

Zhou Wang [1], Yijing Lin [1], Yuhang Dai [1], Jiakui Fan [1], Weihong Sun [1], Junyuan Chen [1], Siqi Yang [1], Shiting Dou [1], Haoxiang Zhu [1], Yan Gu [1], Jin Wang [2], Hao Zhang [3], Qiang Chen [1,*], and Xiaoyan Liu [1,*]

[1] College of Integrated Circuit Science and Engineering, Nanjing University of Posts and Telecommunications, Nanjing 210023, China; zhouwang@njupt.edu.cn (Z.W.); b22030404@njupt.edu.cn (Y.L.); 2022221004@njupt.edu.cn (Y.D.); b23030720@njupt.edu.cn (J.F.); b23030312@njupt.edu.cn (W.S.); 1023223316@njupt.edu.cn (J.C.); 1224228617@njupt.edu.cn (S.Y.); 1224228412@njupt.edu.cn (S.D.); 1223228218@njupt.edu.cn (H.Z.); yangu@njupt.edu.cn (Y.G.)
[2] College of Electronic and Optical Engineering & College of Flexible Electronics (Future Technology), Nanjing University of Posts and Telecommunications, Nanjing 210023, China; jin@njupt.edu.cn
[3] Suzhou Laboratory, Suzhou 215000, China; zhangh@szlab.ac.cn
* Correspondence: 20230222@njupt.edu.cn (Q.C.); xiaoyanliu@njupt.edu.cn (X.L.)

Table S1. Results of total attenuation coefficient versus wavelength for pure seawater at 100 m communication distance.

**Wavelength (nm)**	**440**	**475**	**490**	**505**	**515**	**525**
$\eta(\lambda)$	1.89×10^{-2}	1.98×10^{-2}	2.25×10^{-2}	3.32×10^{-2}	4.40×10^{-2}	5.18×10^{-2}
SNR	30.00	29.61	28.46	23.81	19.11	15.71
BER	4.72×10^{-4}	5.90×10^{-4}	1.00×10^{-3}	1.83×10^{-3}	2.36×10^{-3}	2.95×10^{-3}

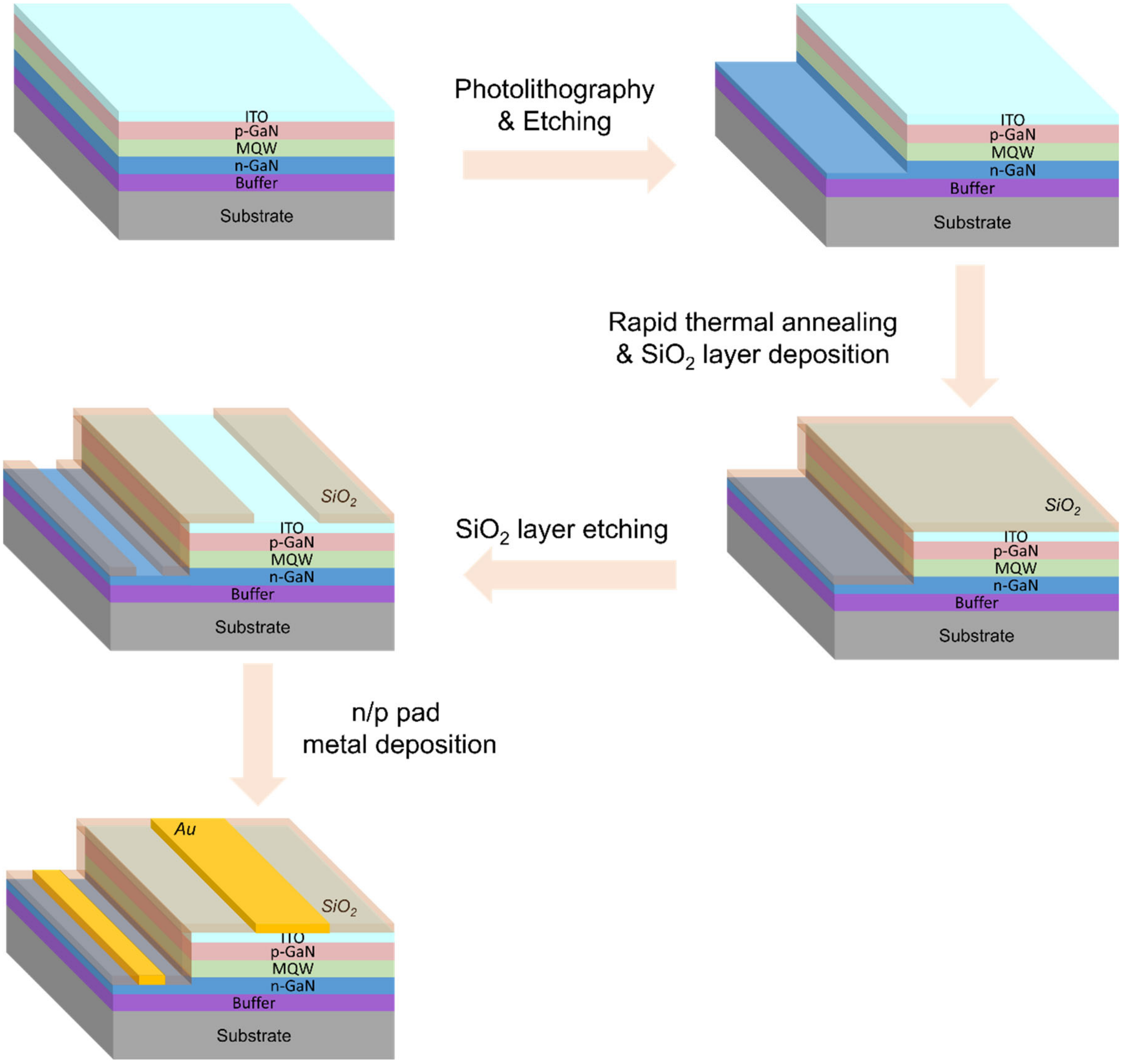

Figure S1. The typical manufacturing process of GaN-based micro-LED.

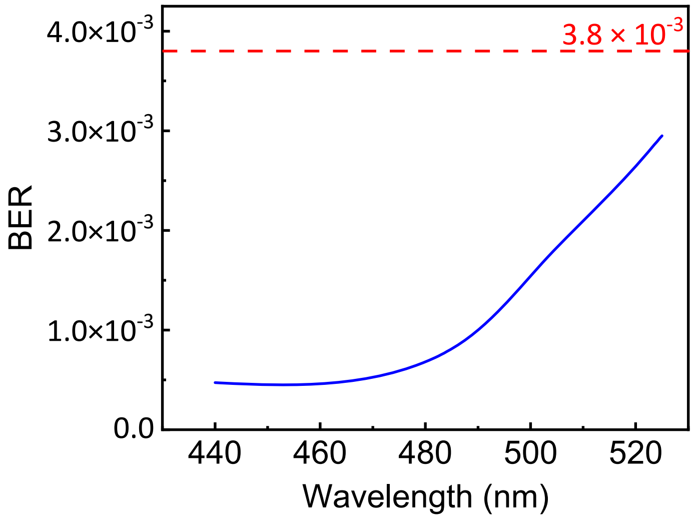

Figure S2. Variation of total attenuation coefficient with wavelength for pure seawater at 100 m communication distance.
